# Intestinal parasites among intellectually disabled individuals in Iran: a systematic review and meta-analysis

**DOI:** 10.1186/s13099-021-00424-6

**Published:** 2021-05-01

**Authors:** Mohammad Javad Abbaszadeh Afshar, Mehdi Mohebali, Sina Mohtasebi, Aref Teimouri, Bahareh Sedaghat, Reza Saberi

**Affiliations:** 1 Department of Medical Parasitology and Mycology, School of Medicine, Jiroft University of Medical Sciences, Jiroft, Iran; 2 Department of Medical Parasitology and Mycology, School of Public Health, Tehran University of Medical Sciences, Tehran, Iran; 3 Department of Parasitology and Mycology, School of Medicine, Shiraz University of Medical Sciences, Shiraz, Iran; 4 Toxoplasmosis Research Center, Department of Parasitology, School of Medicine, Mazandaran University of Medical Sciences, Sari, Iran

**Keywords:** Intestinal parasites, Intellectual disability, Mental retardation, Systematic review, Meta-analysis, Iran

## Abstract

**Background:**

Poor self-care skills and personal hygiene resulted from limitations in learning and understanding, put intellectually disabled individuals at greater risk for intestinal parasitic infections (IPIs). Despite several regional reports in Iran, the overall burden on IPIs among intellectually disabled individuals is poorly understood. Hence, the present study aimed to estimate the pooled prevalence of IPIs among intellectually disabled individuals in Iran.

**Methods:**

Using the PRISMA guidelines, we conducted a systematic review and meta-analysis of data retrieved from seven electronic databases (PubMed, Web of Science, Scopus, Embase, and ProQuest for English articles, as well as SID and Magiran for Persian) from their inception up to December 2020. Pooled prevalence was estimated using a random-effects model with a 95% confidence interval (CI) and depicted as a forest plot, while heterogeneity was evaluated using Cochran’s Q-test.

**Results:**

Exactly 1263 of the 3004 intellectually disabled individuals examined by 14 studies across 10 provinces of Iran were positive for IPIs. Overall pooled prevalence estimate was 41% (95% CI 29–53%) with a range of 21% (95% CI 10–32%) to 68% (95% CI 55–80%) across sub-groups. *Entamoeba coli* (16.2%; 95% CI 10.3–22%), *Blastocystis* spp. (12.2%; 95% CI 7.2–17.2%), and *Giardia duodenalis* (11.9%; 95% CI 7.4–16.3%) were the most prevalent protozoan species. In terms of helminthic agents, the most prevalent species were *Enterobius vermicularis* (11.3%; 95% CI 6.3–16.3%) followed by *Strongyloides stercoralis* (10.9%; 95% CI 5.0–16.9%) and *Hymenolepis nana* (2.8%; 95% CI 0.4–5.2%)

**Conclusion:**

IPIs are highly prevalent among intellectually disabled individuals in Iran. Improving the health status and implementing infectious disease prevention strategies in rehabilitation centers, health promotion interventions to improve personal hygiene of intellectually disabled individuals, as well as utilize sensitive diagnostic methods besides routine stool examination techniques, and treatment of infected individuals will help in the control of these infections among intellectually disabled individuals.

**Supplementary Information:**

The online version contains supplementary material available at 10.1186/s13099-021-00424-6.

## Background

Intestinal parasitic infections (IPIs) are among the leading causes of global health problems, especially among the deprived communities where poor personal hygiene, environmental sanitation, socio-economic, demographic, and health-related behaviors have contributed to a notable prevalence. *Giardia duodenalis*, *Entamoeba histolytica*, *Cryptosporidium* spp., and *Ascaris lumbricoides* play significant roles in this scenario. However, parasites such as *Enterobius vermicularis* and *Strongyloides stercoralis* are still ignored [1, 2].

In the case of IPIs, the vulnerabilities of certain groups of people have been highlighted, such as children, immunocompromised patients, and intellectually disabled individuals. Among these, less apparent has been the plight of people with intellectual disability, who have a range of vulnerabilities that include health problems, mental disorders, and social disadvantage [3]. Intellectual disability, formerly defined as mental retardation, is a cluster of disorders characterized by low intelligence and associated limitations in adaptive behavior [4]. Most people with such disabilities cannot be trained for proper health behaviors and are prone to get the infection. Due to limitations in learning and understanding, poor self-care skills, poor personal hygiene, and pica habits, intellectually disabled individuals are at higher risk of IPIs. In addition, people with intellectual disabilities are mostly kept in crowded places such as rehabilitation centers or care homes for a long time, where puts them at a greater risk for IPIs [5, 6].

In a resource-limited country like Iran, with about 80 million people, cost-effectiveness in the control of IPIs is essential to ensure efficient allocation of resources and achievement of high impact. Hence, investigation and providing epidemiological information on IPIs among intellectually disabled individuals, as a high-risk population, could help to design a targeted and cost-effective control program. Despite several regional reports in Iran, the overall burden on IPIs among intellectually disabled individuals is poorly understood. Hence, the present systematic review and meta-analysis, which is the first of its kind, aimed to estimate the pooled prevalence of IPIs among intellectually disabled individuals in Iran. This will serve as a guide for targeted control and ensure cost-effective control of IPIs in Iran.

## Methods

The present study followed the preferred reporting items for systematic reviews and meta-analysis (PRISMA) guideline published by Moher et al. [7], and the inclusion of data for quantitative synthesis was entered, based on the PRISMA checklist (Additional file 1). The infection of intellectually disabled individuals with intestinal parasites in Iran was the outcome of interest.

### Search strategy

Published studies searched in five international databases (PubMed, Web of Science, Scopus, Embase, and ProQuest) and two national databases (SID and Magiran) from their inception up to December 2020 (Additional file 2). Furthermore, the Google Scholar database was used for proofing the search. Relevant articles were found using the following search terms: (‘intestinal parasites’ or ‘parasitic intestinal disease’ or ‘intestinal protozoa’ or ‘intestinal helminths’ or ‘soil-transmitted helminth’) and (‘intellectually disabled’ or ‘mentally disabled’ or ‘mentally retarded’ or ‘rehabilitation center’) and (‘Iran’). Bibliographic lists of the relevant studies were searched to find other associated articles that were not found through database searching. All searched articles were imported to EndNote X8 software (Thompson Reuter, CA, USA) for management.

### Eligibility criteria

Eligibility for the inclusion of each study was based on the following conditions: (a) it was carried out in Iran, (b) it was reported IPIs among intellectually disabled individuals, (c) it was published in English or Persian, (d) it was a cross-sectional study, (e) sample size and the number of positive cases were clearly stated, (f) it was published in a peer-reviewed journal, and (g) parasites were identified at least to the genus level. Studies that did not meet these inclusion criteria were excluded.

### Study selection and data extraction

After removing duplicated articles, the studies screened through title and abstract for relevance. Then, a full-text review to determine the presence of the inclusion requirements. To ensure data validation and increase the likelihood of detecting errors, literature search, screening of articles, article selection for eligibility, and data extraction were performed by two authors independently. Also, discrepancies were removed following discussion with the third reviewer. Data pulled out from each included study, where the author names, the year the study was carried out, the year of the publication, sample size, number of positive cases, study location (region/province), type of the reported intestinal parasites, and method of diagnosis.

### Quality assessment

Quality assessment of the included studies was carried out using the Joanna Briggs Institute (JBI) critical appraisal instrument for studies reporting prevalence data [8] (Additional file 3). This tool comprised nine items with four options include ‘yes’, ‘no’, ‘unclear’, and ‘not applicable’. The ‘yes’ answers were used to calculate the final score of each article. Answers to the mentioned questions for individual studies were respectively assigned scores of 0 or 1 for ‘yes’ or ‘no’ answers, while ‘unclear’ or ‘not applicable’ were used when a study does not clearly answer the question or when the question was not applicable for the study. For study inclusion in the quantitative synthesis, a minimum quality assessment score of 6 (66.7%), which means answering ‘yes’ to at least six of the nine questions on the checklist was required.

### Data synthesis and statistical analysis

Preliminary analyses, including summations, subtractions, divisions, multiplications, and estimation of percentages, were carried out using Microsoft Excel (Microsoft^®^ Office 2013). Statistical and meta-analyses were conducted using Stata^®^ statistical software version 14 (Stata Corp, College Station, TX, USA).

### Pooling, sub-group, and heterogeneity analyse^s^

Pooled prevalence and their 95% confidence interval (CI) were estimated by the random-effects model and presented as a forest plot [9]. Sub-group analysis was carried out based on the geographical regions, study period (year/s of conduction), sample size, diagnostic methods, risk of bias, and type of the reported intestinal parasites (protozoa/helminth).

Heterogeneity, which is the measure of variability between studies analyzed, was evaluated using Cochran’s Q-test, while percentage variation in prevalence estimate due to heterogeneity, was quantified using the inverse variance (I^2^) statistic. I^2^ values of 0, 25, 50, and 75% were considered as ‘no’, ‘low’, ‘medium’, and ‘high’ heterogeneities, respectively [10].

### Publication bias, sensitivity, and meta-regression analyses

Publication bias (across-study bias) was examined by funnel plots, while the statistical significance was assessed by the Egger’s regression asymmetry test [11] and Begg’s rank correlation methods [12], respectively. A sensitivity analysis was carried out using a random-effect model through step-by-step omitting of a single study to evaluate the robustness of the pooled prevalence estimate [13]. Moreover, meta-regression was carried out by considering the publication year and sample size to detect the potential source of heterogeneity.

## Results

### Literature search and eligible studies

**Fig. 1:**
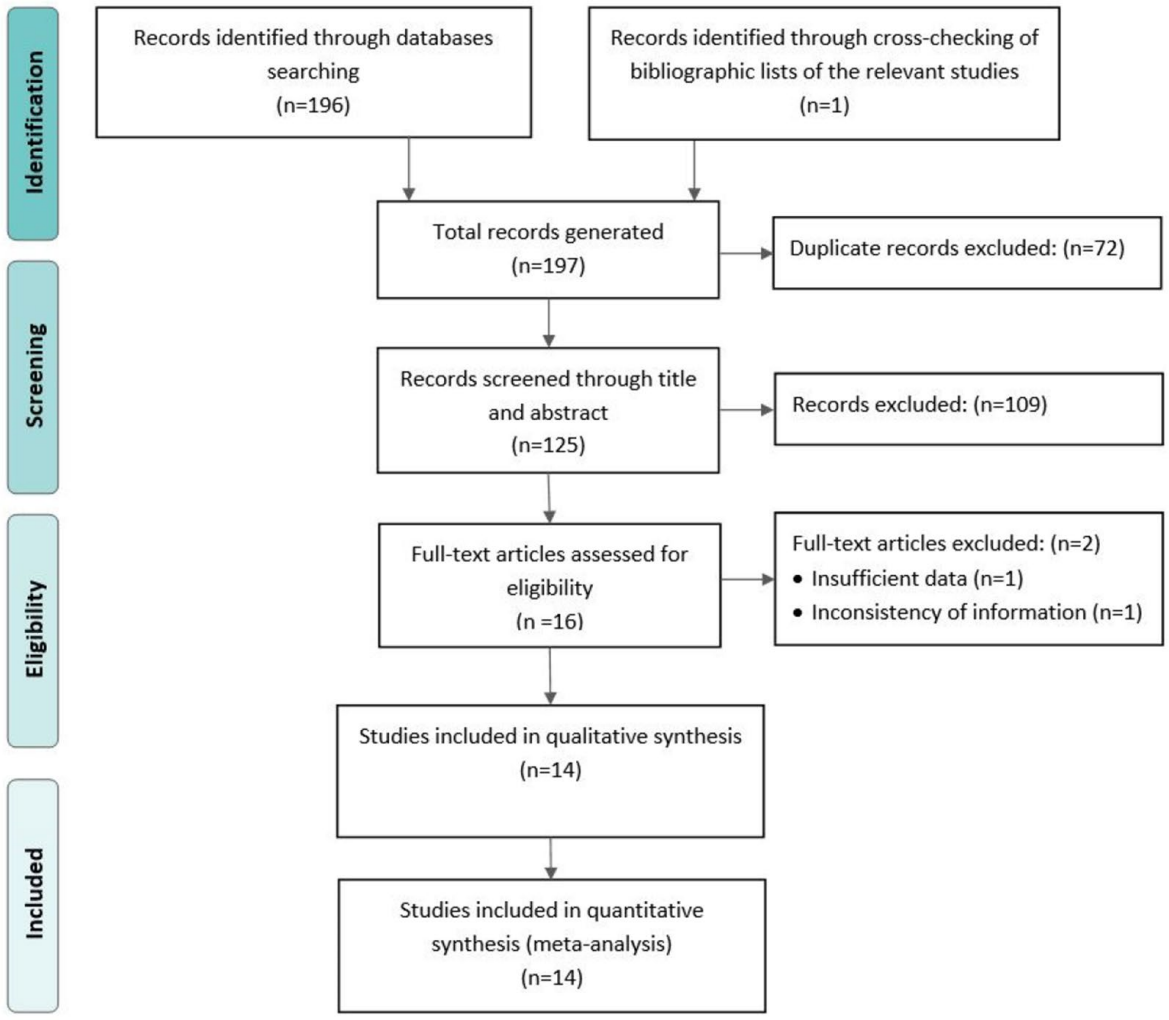
PRISMA flowchart diagram for the selection process of eligible studies

The procedure for the selection of eligible studies is presented in Fig. 1. Of the 197 studies identified, 196 were retrieved through the search of databases and one from the lists of eligible article references. Seventy-two studies were excluded because of duplication. One hundred-twenty-five studies were subjected to title and abstract review, where 109 studies were excluded. Full text of 16 remains studies assessed, and two studies excluded because of the inconsistency in the information (n = 1) and insufficient data on sample sizes and the number of cases (n = 1). Finally, 14 studies were subjected to quantitative synthesis.

### Characteristics of the eligible studies

Table 1 shows the characteristics of the eligible studies. Fourteen studies examined 3004 intellectually disabled individuals for IPIs in Iran and reported prevalence rates ranging between 5.2 and 79%. Studies were conducted between 1991 and 2017 and published between 1994 and 2019. Three studies utilized the Graham test for *E. vermicularis* diagnosis. Also, three studies utilized agar plate culture for S*. stercoralis* and hookworm diagnosis. Seven studies had a sample size of less than 200, while only three studies had a sample size of more than 300. None of the studies assessed for quality by the JBI critical appraisal instrument was excluded for lack of merit. Quality scores ranged between 6 and 8 (66.67–88.89%) of a total of 9 scores (Table 1 and Additional file 4).

**Table 1:** List and characteristics of the 14 eligible studies for IPIs among intellectually disabled individuals in Iran, 2020

No.	Authors (Reference)	Year of study	Publication year	Province	Region	Sample size	No. of positive	Prev. (%)	Diagnostic method	QAS
1	Rouhani et al. [31]	1991/1992	1994	Tehran	Center	330	196	59.4	D-F-G	7
2	Mahyar et al. [32]	1998	2000	Qazvin	Center	258	146	56.6	D-F	7
3	Mousavian et al. [33]	2004	2006	Tehran	Center	175	113	64.6	D-F-G	8
4	Sharif et al. [34]	2008	2010	Mazandaran	North	362	95	26.2	D-F-S	7
5	Hazrati Tappeh et al. [35]	2007/2008	2010	Urmia	North-west	225	46	20.4	D-F	8
6	Davari et al. [36]	2011	2011	Ardabil	North-west	216	95	44.0	D-F-S	7
7	Rasti et al. [37]	2006/2007	2012	Isfahan	Center	243	192	79.0	D-F-G	7
8	Shokri et al. [29]	2010	2012	Hormozgan	South	133	64	48.1	D-F-S	6
9	Soosaraei et al. [38]	2009	2014	Golestan	North	196	24	12.2	D-F-S	7
10	Anvari et al. [39]	2014	2015	Yazd	Center	129	55	42.6	D-F-S	6
11	Ahmadi et al. [40]	2013/2014	2015	Mazandaran	North	341	112	32.8	D-F-S-C	7
12	Soleimani et al. [41]	2015	2016	Mazandaran	North	97	5	5.2	D-F	7
13	Saeidnia et al. [42]	2013	2016	Gilan	North	173	51	29.5	D-F-S-C	8
14	Mohammadi et al. [43]	2016/2017	2019	Hormozgan	South	126	69	54.8	D-F-S-C	7

D: Direct wet-mount; F: Formalin-ether; S: Staining (Trichrome and Ziehl–Neelsen); G: Graham test; C: Agar plate culture; Prev.: Prevalence; QAS: Quality assessment score

### Pooling, sub-group, and heterogeneity analyses

**Fig. 2:**
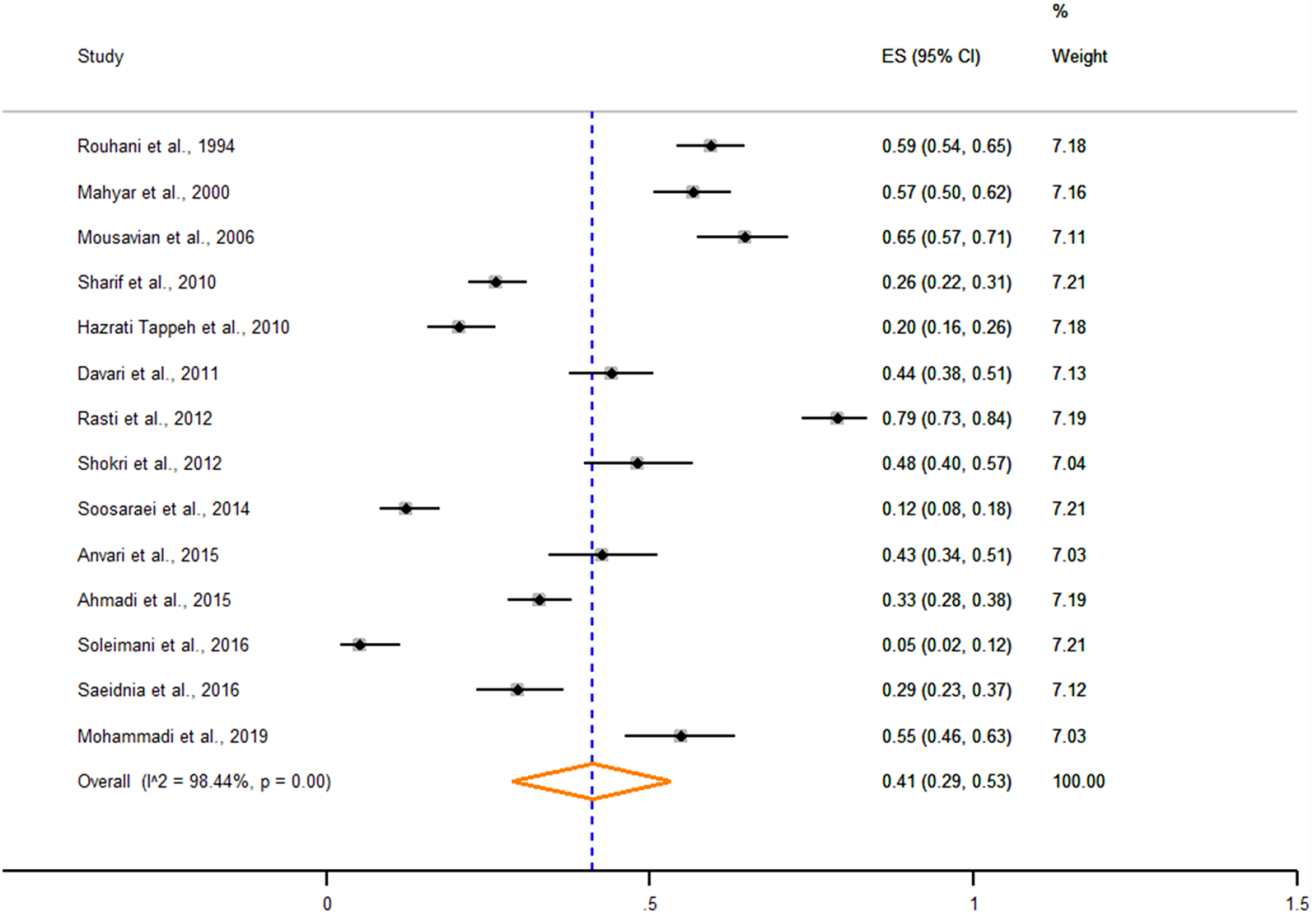
Forest plot showing the pooled prevalence of IPIs among intellectually disabled individuals in Iran, 2020

Fourteen studies reported 1263 positive cases of IPIs among 3004 intellectually disabled individuals examined from ten provinces of Iran. The overall pooled prevalence of the IPIs among intellectually disabled individuals was estimated at 41% (95% CI 29–53%) (Fig. 2). Pooled prevalence estimates for subgroups, including geographical region, study period, sample size, diagnostic method, and risk of bias, are presented in Table 2. The highest prevalence estimates were recorded in center regions (61%; 95% CI 49–72%). In terms of the diagnostic method, studies that implemented the Graham test, besides direct wet-mount and concentration methods, recorded the highest pooled prevalence estimate (68%; 95% CI 55–80%). Also, high heterogeneity was observed in the results of the included studies (Q = 833.9, I^2^ = 98.4%, and *p* < 0.001).

**Table 2:** Pooled prevalence estimates for IPIs among intellectually disabled individuals stratified according to sub-groups in Iran, 2020

Variables	No. of studies	Pooled prevalence estimates			Heterogeneity	
		Sample size	Positives	Prevalence (95% CI)	*I*^*2*^ (%)	*Q-p*
Region						
Center	5	1135	596	61 (49–72)	93.9	< 0.001
North	5	1169	430	21 (10–32)	95.7	< 0.001
North-east	2	441	117	30 (25–34)	–	–
South	2	259	120	51 (45–57)	–	–
Study period						
1991–2000	2	588	342	58 (54–62)	–	–
2001–2010	6	1334	605	42 (19–64)	98.9	< 0.001
2011–2020	6	1082	316	35 (19–50)	97.1	< 0.001
Sample size						
< 200	7	1029	403	37 (19–54)	98.4	< 0.001
200–300	4	942	479	50 (24–76)	99.1	< 0.001
300 <	3	1033	403	39 (20–59)	98.8	–
Diagnostic method						
D-F	3	580	197	27 (0–56)	–	–
D-F-G	3	748	501	68 (55–80)	–	–
D-F-S	5	1036	333	34 (21–48)	96.0	< 0.001
D-F-S-C	3	640	232	39 (26–52)	–	–
Risk of bias						
Low	9	2123	884	42 (27–56)	98.3	< 0.001
Medium	5	881	379	40 (16–64)	98.7	< 0.001
Overall	14	3004	1263	41 (29–53)	99.8	< 0.001

D: Direct wet-mount; F: Formalin-ether; S: Staining (Trichrome and Ziehl–Neelsen); G: Graham test, C: Agar plate culture; CI: Confidence interval; I^2^: Inverse variance index; Q-*p*: Cochran’s *p*-value

### Intestinal parasites species-specific pooled prevalence

For intestinal protozoa, *Entamoeba coli* (16.2%; 95% CI 10.3–22%), *Blastocystis* spp. (12.2%; 95% CI 7.2–17.2%), and *G. duodenalis* (11.9%; 95% CI 7.4–16.3%) were the most prevalent species. In terms of helminthic agents, the most prevalent species were *E. vermicularis* (11.3%; 95% CI 6.3–16.3%) followed by *S. stercoralis* (10.9%; 95% CI 5.0–16.9%) and *Hymenolepis nana* (2.8%; 95% CI 0.4–5.2%). Species-specific pooled prevalence estimates for IPIs among intellectually disabled individuals in Iran are embedded in Table 3.

**Table 3:** Species-specific pooled prevalence estimates for IPIs among intellectually disabled individuals in Iran, 2020

Parasite	No. of studies	Pooled prevalence estimates			Heterogeneity	
		Sample size	Positives	Prevalence (95% CI)	*I*^*2*^ (%)	*p*
Protozoa						
*Entamoeba coli*	13	2829	495	16.2 (10.3–22.0)	96.4	< 0.001
*Blastocystis* spp.	10	1900	248	12.2 (7.2–17.2)	94.8	< 0.001
*Giardia duodenalis*	12	2566	329	11.9 (7.4–16.3)	94.4	< 0.001
*Iodamoeba butschlii*	8	1868	84	3.9 (2.2–5.6)	76.1	< 0.001
*Chilomastix mesnili*	7	1734	84	3.6 (1.5–5.7)	92.2	< 0.001
*Endolimax nana*	6	1370	51	2.9 (0.9–5.0)	88.0	< 0.001
*Entamoeba histolytica/dispar*	7	1830	69	2.6 (1.0–4.2)	89.3	< 0.001
*Dientamoeba fragilis*	1	243	4	–	–	–
*Entamoeba hartmanni*	1	243	58	–	–	–
Helminths						
*Enterobius vermicularis*	8	1686	234	11.3 (6.3–16.3)	97.5	< 0.001
*Strongyloides stercoralis*	5	1103	112	10.9 (5.0–16.9)	95.6	< 0.001
*Hymenolepis nana*	4	847	33	2.8 (0.4–5.2)	83.7	< 0.001
*Trichuris trichiura*	4	984	24	2.0 (0.7–3.2)	52.2	0.09
*Ascaris lumbricoides*	2	588	7	1.2 (0.3–2.1)	–	–
*Dicrocoelium dendriticum*	2	599	28	0.9 (0.1–1.6)	–	–
*Taenia* spp.	2	671	3	0.4 (0.0–0.9)	–	–
*Trichostrongylus* spp.	2	599	3	0.4 (0.1–0.9)	–	–
Hookworms	1	330	1	–	–	–

CI: Confidence interval; I^2^: Inverse variance index

### Publication bias, sensitivity, and meta-regression analyses

**Fig. 3:**
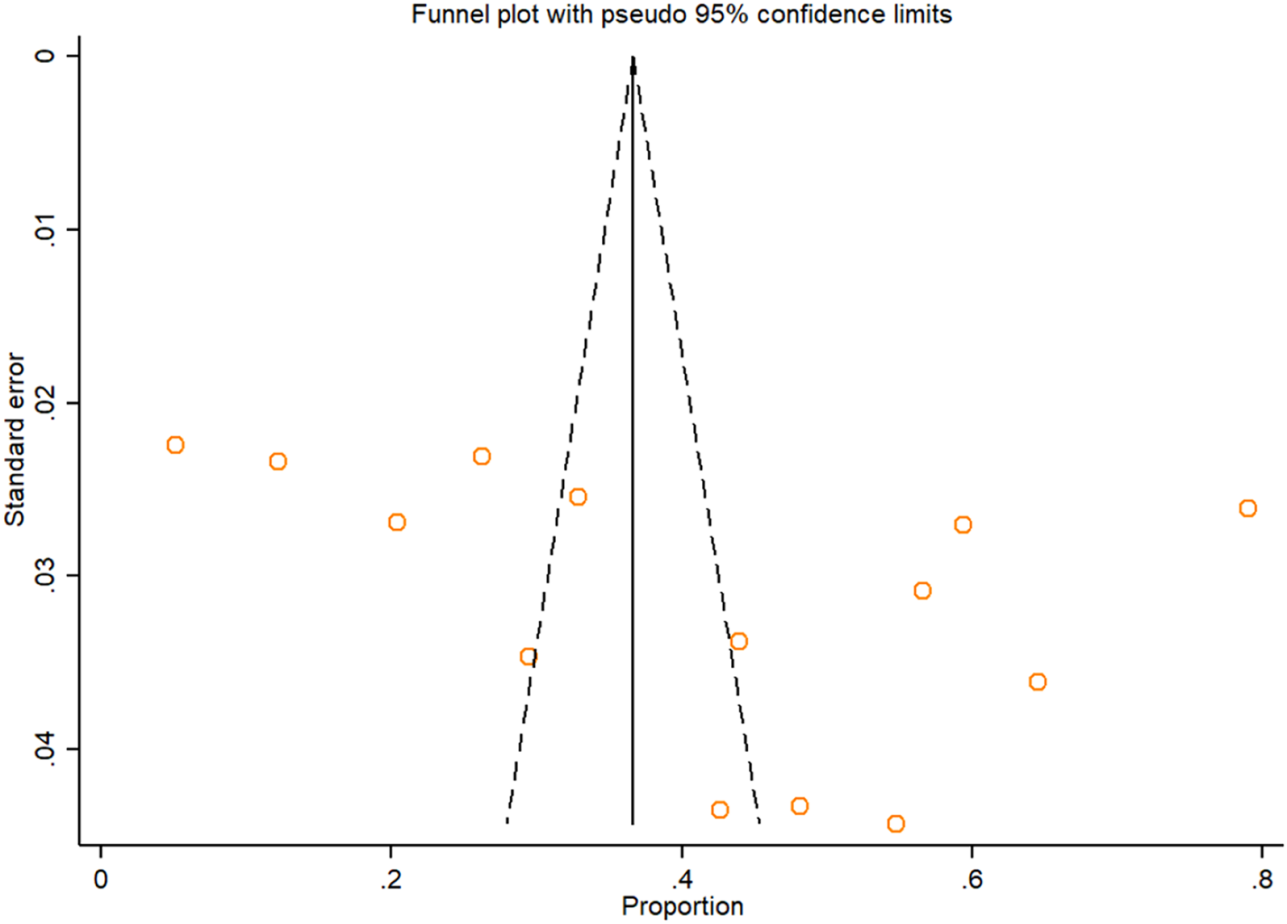
Funnel plot displaying the prevalence estimate of IPIs among intellectually disabled individuals in Iran, 2020

**Fig. 4:**
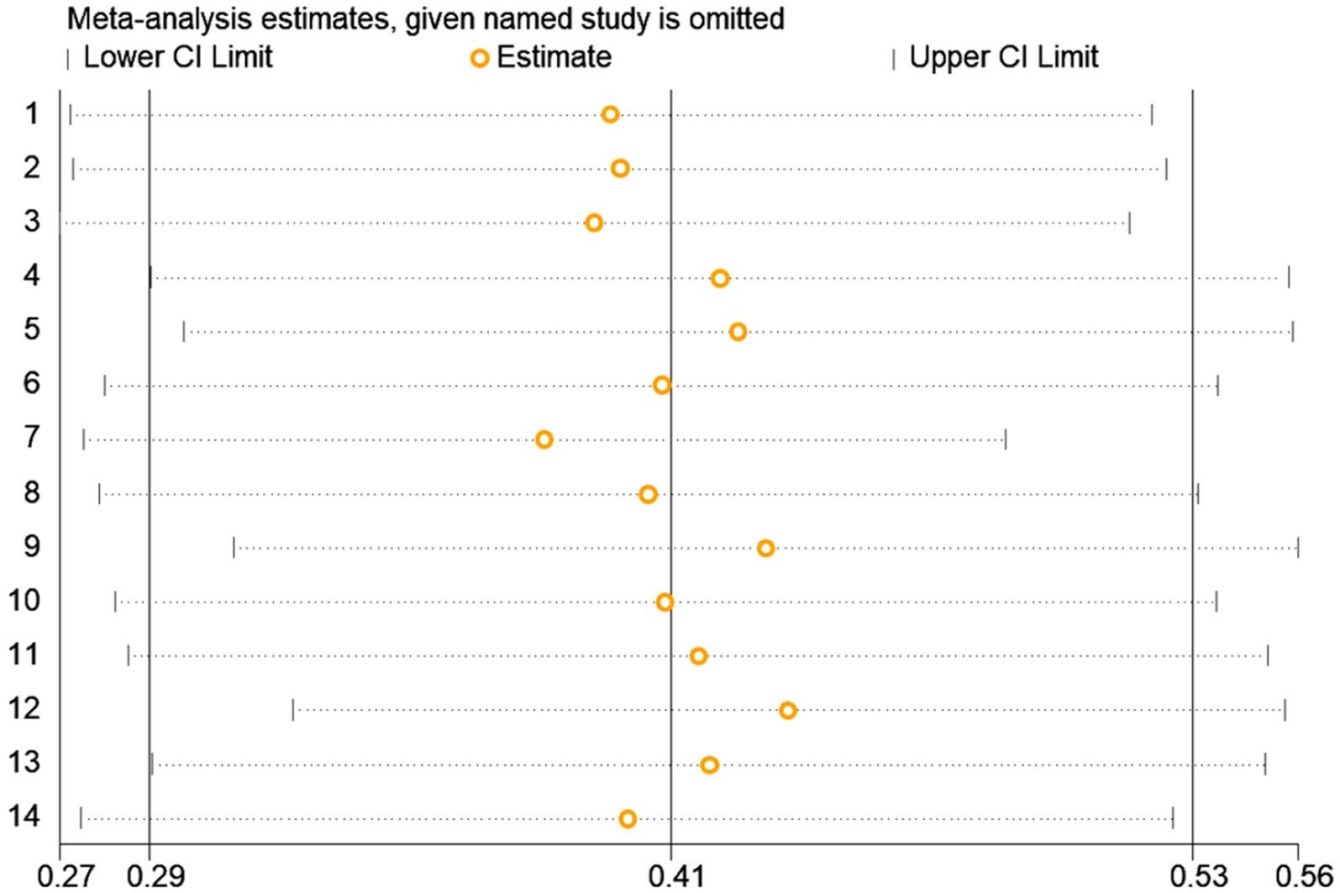
Sensitivity analysis of included studies to determine the pooled prevalence estimate of IPIs among intellectually disabled individuals in Iran, 2020 (1 to 14: The included studies, in the order embedded in Table 1)

Symmetrical funnel plot visual inspection (Fig. 3) showed absence of publication bias, which was statistically verified by Begg’s test (*p* = 0.112) and Egger’s test (bias coefficient (B) = 15.79 (95% CI − 3.98–35.56; *p* = 0.107). Sensitivity analysis was carried out by excluding each study step-by-step from the meta-analysis and comparing the point prevalence estimates before and after removing a single study. Removing a single study did not alter the pooled prevalence estimate considerably, with sensitivity analysis ranging from 38% (when study No.7 was removed) to 44% (when study No.12 was removed) (Fig. 4). A univariate meta-regression between the infection prevalence and the year of publication and the sample size showed no statistically significant correlations (*p* > 0.05) (Table 4).

**Table 4:** Univariate meta-regression of factors related to the heterogeneity of IPIs among intellectually disabled individuals in Iran, 2020

Variables	Coefficient	*p*-value
Year of publication	− 0.0129388	0.143
Sample size	0.0003108	0.672

## Discussion

An adequate understanding of the nationwide burden of IPIs and the widespread species, particularly in high-risk populations is essential for targeted and cost-effective prevention and control. Thus, the current systematic review and meta-analysis is the first of its kind in Iran that provides comprehensive useful epidemiological information regarding IPIs among intellectually disabled individuals that may serve as a guide for disease control in Iran.

We observed an overall pooled prevalence of 41% among intellectually disabled individuals which is more than 38% and 19% for Iranian school children [14] and food handlers [15], respectively. The population density in rehabilitation centers and care homes, close contact, poor personal hygiene due to limitation in learning, lack of health facilities, as well as lack of sufficient knowledge of the authorities managing rehabilitation centers on intestinal parasites transmission modes may explain the high prevalence of IPIs among intellectually disabled individuals in Iran. On the other hand, as intellectually disabled individuals have difficulties in communications, and it is not easy for them to express their gastrointestinal discomforts and complaints, many IPIs harm them chronically.

Though there are no available systematic reviews and meta-analyses conducted on related topics in Iran and elsewhere, the pooled prevalence of IPIs among intellectually disabled individuals in the present study is higher than the primary studies conducted in Tanzania (12.4%) and Italy (23%) [16, 17]. However, the pooled prevalence estimate of IPIs among intellectually disabled individuals in the present study is lower than the primary study conducted in Egypt (43.5%) [18]. These variations may be attributable to differences in the levels of hygiene and sanitation and health education among different rehabilitation centers as well as the specificity and sensitivity of the diagnostic methods employed by the individual studies.

Rehabilitation center staff and their families are at risk of infection due to their direct/indirect contact with intellectually disabled individuals. In this case, Barazesh et al. [19] reported a significant prevalence (34%) of IPIs among the staff of a rehabilitation center in the north-east of Iran. Regarding the high prevalence of IPIs among intellectually disabled individuals in the present study, it is crucial for informing the staff about transmission modes of intestinal parasites.

Sub-group analysis based on the study period revealed a 23% decline in the prevalence of IPIs among intellectually disabled individuals during the past three decades in Iran. Although due to the implementation of a non-targeted control strategy in Iran, the prevalence of IPIs has a dramatic decrease in children [14], these infections remain a challenging public health problem in intellectually disabled individuals.

*E. coli*, *Blastocystis* spp., and *G. duodenalis*, were the most predominant protozoan species reported during the period under review. These species are the most commonly reported in Iran in almost all studies on IPIs among different populations [2, 20]. The majority of individuals infected with these species are asymptomatic and excrete numerous cysts that remain viable for a long time in the environment. It seems the healthy carrier, an environmentally resistant cyst, which enhances the chance of these species to infect the new host, as well as a great potential to transmit as a water/foodborne infection [15, 21, 22], could explain the high prevalence of these species compared with other protozoan species.

*E. vermicularis* and *S. stercoralis* following *H. nana* were the most predominant helminth records among intellectually disabled individuals in Iran. Institutions for intellectually disabled individuals have been reported to be hyper-endemic for several helminths, such as *S. stercoralis* and *E. vermicularis* (24). The high prevalence of these species could be attributable to the fact that these species can easily be spread in crowded places, such as rehabilitation centers and their dormitories where the person-to-person is the main mode of intestinal parasites transmission due to close contact. On the other hand, autoinfection, as a hallmark of enterobiasis, strongyloidiasis, and hymenolepiasis, could cause protracted infection in intellectually disabled individuals [23–25]. Although the prevalence of human helminthic diseases has plunged in recent decades throughout Iran, some of them, particularly those with direct fecal–oral transmissions, such as *Enterobius* and *Hymenolepis*, remain common [26]. The high prevalence of these parasites among intellectually disabled individuals should not be considered a sudden infection and seems to be due to the previous, chronic, accumulated, and untreated infections.

In the present study, the highest prevalence estimates were recorded in central regions. Urbanization and subsequently more tendency to keeping intellectually disabled individuals in rehabilitation centers and their crowded dormitories may explain the high prevalence of IPIs in the central region of Iran. However, *S. stercoralis*, as a soil-transmitted helminth, was mostly reported from the northern (Mazandaran and Gilan) and southern (Khuzestan, Bushehr, and Hormozgan) regions [27–29], which are considered the humid areas in Iran.

The higher pooled prevalence estimates in the studies which employed the Graham test and agar plate culture besides routine stool examinations reflect the fact that implementation of these methods together enhanced the sensitivity of intestinal parasites detection [30]. Since the routine stool examination methods such as direct and concentration techniques, have low sensitivity for detection of *E. vermicularis* and *S. stercoralis* and due to the high prevalence of these two nematodes in the rehabilitation centers, additional tests such as the Graham test for *E. vermicularis* and agar plate culture and serological methods for *S. stercoralis* should be considered in the periodic medical check-ups of intellectually disabled individuals.

As the public health implications of our findings, the present epidemiological information could be a springboard for more investigation of IPIs among intellectually disabled individuals and subsequently designing a targeted control program to reduce the burden of IPIs in Iran. For this purpose, further direct interventions are suggested as follows: (a) health promotion interventions to improve personal hygiene among intellectually disabled individuals, besides implementing infectious disease prevention strategies in rehabilitation centers, (b) informing staff about how intestinal parasites are transmitted among intellectually disabled individuals and between them and the staff, (c) periodic check-ups of intellectually disabled individuals and rehabilitation centers staffs for IPIs, (d) regarding the high prevalence of *E. vermicularis* and *S. stercoralis* in rehabilitation centers, considering sensitive diagnostic methods such as Graham test, and serological and stool culture methods, and (e) allocate sufficient funds by health policymakers to prevent and control IPIs in rehabilitation centers. Also, we suggest more studies using sensitive diagnostic methods in rehabilitation centers of different provinces such as Khorasan, Sistan and Baluchistan, and Kerman provinces.

## Limitations

Though this study provided valuable epidemiological information on the prevalence of IPIs among intellectually disabled individuals in Iran, which will be useful in disease control, it is not devoid of limitations. We could not include some potentially relevant studies which would have added to the understanding of IPIs among intellectually disabled individuals due to insufficiency of data. Some regions (east, north-east, and south-east) were not represented in the analysis because no study was published from these regions. Another setback is the study revealed a high heterogeneity among studies, which may be due to variations in sample populations, region, and diagnostic methods employed by the various studies.

## Conclusion

IPIs are highly prevalent among intellectually disabled individuals in Iran with an overall pooled prevalence estimate of 41%. However, there was a 23% decline in the pooled prevalence over 26 years, but IPIs in this high-risk population remain substantial. In addition to *E. coli*, *G. duodenalis*, and *Blastocystis* spp. as prevalent protozoan species, the prevalence of *E. vermicularis* and *S. stercoralis* were significant in intellectually disabled individuals. Besides taking into account the presented epidemiological information, rehabilitation centers authorities should adopt a responsible approach, including improving the health status of rehabilitation centers through implementing infectious disease prevention strategies and providing sanitary facilities. Health education and awareness about intestinal parasites transmission routes in rehabilitation centers, utilizing sensitive diagnostic methods besides routine stool examination techniques, as well as treatment of infected individuals, will help in the control of IPIs among intellectually disabled individuals.

## Supplementary Information

**Additional file 1.** PRISMA Checklist.

**Additional file 2.**  Search strategy.

**Additional file 3.** JBI Critical Appraisal Checklist for Studies Reporting Prevalence Data.

**Additional file 4.** Quality assessment scores for eligible studies.

## Data Availability

All data generated or analyzed during this study are included in this published article and its Additional files

## Abbreviations

IPIs: Intestinal parasitic infections
PRISMA: Preferred reporting items for systematic reviews and meta-analysis
SID: Scientific Information Database
JBI: Joanna Briggs Institute
CI: Confidence interval
I^2^: Inverse variance
