# Supplementary material for: Intestinal parasites among intellectually disabled individuals in Iran: a systematic review and meta-analysis
Source: Gut Pathog. 2021 May 1;13:28. doi: 10.1186/s13099-021-00424-6 (PMC8088632; doi:10.1186/s13099-021-00424-6)
Supplement: Supplementary file 2 — Additional file 2. Search strategy. [file 13099_2021_424_MOESM2_ESM.docx]

| Additional file 2. Literature Search strategies for IPIs among intellectually disabled individuals in Iran, 2020 | | |
| --- | --- | --- |
| Database | **Search strategy** | **Search results** |
| International databases | | |
| PubMed | ((("intestinal parasites"[Title/Abstract] OR "intestinal protozoa"[Title/Abstract] OR "intestinal helminths"[Title/Abstract] OR "parasitic intestinal disease"[Title/Abstract] OR "soil transmitted helminth"[Title/Abstract]) AND "mentally disabled"[Title/Abstract]) OR "intellectually disabled"[Title/Abstract] OR "mentally retarded"[Title/Abstract] OR "rehabilitation center"[Title/Abstract]) AND "Iran"[Title/Abstract] | 52 |
| Web of Science | TOPIC: (Intestinal parasites OR Intestinal protozoa OR Intestinal helminths OR Parasitic Intestinal Disease OR Soil transmitted helminth) AND TOPIC: (Mentally disabled OR Intellectually Disabled OR Mentally retarded OR Rehabilitation center) AND TOPIC: (Iran) | 5 |
| Scopus | ( ( TITLE-ABS-KEY ( "Intestinal parasites" ) ) OR ( TITLE-ABS-KEY ( "Intestinal protozoa" ) ) OR ( TITLE-ABS-KEY ( "Intestinal helminths" ) ) OR ( TITLE-ABS-KEY ( "Parasitic Intestinal Disease" ) ) ) AND ( ( TITLE-ABS-KEY ( "Mentally disabled" ) ) OR ( TITLE-ABS-KEY ( "Intellectually Disabled" ) ) OR ( TITLE-ABS-KEY ( "Mentally retarded" ) ) OR ( TITLE-ABS-KEY ( "Rehabilitation center" ) ) ) AND ( TITLE-ABS-KEY ( "Iran" ) ) | 6 |
| Embase | ‘intestinal parasites':ab,ti OR 'intestinal protozoa':ab,ti OR 'intestinal helminths':ab,ti OR 'parasitic intestinal disease':ab,ti OR 'soil transmitted helminth':ab,ti AND mentally disabled':ab,ti OR 'intellectually disabled':ab,ti OR 'mentally retarded':ab,ti OR 'rehabilitation center':ab,ti AND iran:ab,ti | 4 |
| National databases | | |
| Scientific Information Database (SID) (In Persian) | (Intestinal parasites) AND (Mentally retarded OR intellectually disabled) | 58 |
| MagIran (In Persian) | (Intestinal parasites) AND (Mentally retarded OR intellectually disabled) | 71 |

Furthermore, the Google Scholar database was used for proofing the search.
