# Supplementary material for: Intestinal parasites among intellectually disabled individuals in Iran: a systematic review and meta-analysis
Source: Gut Pathog. 2021 May 1;13:28. doi: 10.1186/s13099-021-00424-6 (PMC8088632; doi:10.1186/s13099-021-00424-6)
Supplement: Supplementary file 3 — Additional file 3. JBI Critical Appraisal Checklist for Studies Reporting Prevalence Data. [file 13099_2021_424_MOESM3_ESM.docx]

**[Back to top](https://wiki.joannabriggs.org/display/MANUAL/Appendix+5.1:+Critical+Appraisal+Instrument+for+Studies+Reporting+Prevalence+Data#top)**

[**Additional** **file 3:**](https://wiki.joannabriggs.org/display/MANUAL/Appendix+5.1%3A+Critical+Appraisal+Instrument+for+Studies+Reporting+Prevalence+Data) JBI Critical Appraisal Checklist for Studies Reporting Prevalence Data

Reviewer                                                                                         Date

Author                                                               Year                        Record Number

| Questions | Yes | No | Unclear | Not applicable |
| --- | --- | --- | --- | --- |
| 1.         Was the sample frame appropriate to address the target population? | ? | ? | ? | ? |
| 2.         Were study participants sampled in an appropriate way? | ? | ? | ? | ? |
| 3.         Was the sample size adequate? | ? | ? | ? | ? |
| 4.         Were the study subjects and the setting described in detail? | ? | ? | ? | ? |
| 5.         Was the data analysis conducted with sufficient coverage of the identified sample? | ? | ? | ? | ? |
| 6.         Were valid methods used for the identification of the condition? | ? | ? | ? | ? |
| 7.         Was the condition measured in a standard, reliable way for all participants? | ? | ? | ? | ? |
| 8.         Was there appropriate statistical analysis? | ? | ? | ? | ? |
| 9.       Was the response rate adequate, and if not, was the low response rate managed appropriately? | ? | ? | ? | ? |

Overall appraisal:             Include   ?       Exclude   ?       Seek further info?

Comments (Including reason for exclusion)

© Joanna Briggs Institute 2016

Explanation of Prevalence Critical Appraisal

*How to cite:* Munn Z, Moola S, Lisy K, Riitano D, Tufanaru C. (2015) Methodological guidance for systematic reviews of observational epidemiological studies reporting prevalence and incidence data. Int J Evid Based Healthc. 2015; 13:147–153.
