# Supplementary material for: Intestinal parasites among intellectually disabled individuals in Iran: a systematic review and meta-analysis
Source: Gut Pathog. 2021 May 1;13:28. doi: 10.1186/s13099-021-00424-6 (PMC8088632; doi:10.1186/s13099-021-00424-6)
Supplement: Supplementary file 4 — Additional file 4. Quality assessment scores for eligible studies. [file 13099_2021_424_MOESM4_ESM.docx]

**Additional file 4:** Quality assessment scores for eligible studies

| **No.** | **Study Name [Reference No.]** | **Q1** | **Q2** | **Q3** | **Q4** | **Q5** | **Q6** | **Q7** | **Q8** | **Q9** | **Overall score X/9**  **(% Score)** |
| --- | --- | --- | --- | --- | --- | --- | --- | --- | --- | --- | --- |
| 1 | Rouhani et al. [28] | 1 | 1 | 0 | 1 | 1 | 1 | 1 | 1 | NA | 7 (77.8) |
| 2 | Mahyar et al. [29] | 0 | 1 | 1 | 1 | 1 | 1 | 1 | 1 | NA | 7 (77.8) |
| 3 | Mousavian et al. [30] | 1 | 1 | 1 | 1 | 1 | 1 | 1 | 1 | NA | 8 (88.9) |
| 4 | Sharif et al. [31] | 1 | 1 | 0 | 1 | 1 | 1 | 1 | 1 | NA | 7 (77.8) |
| 5 | Hazrati Tappeh et al. [32] | 1 | 1 | 1 | 1 | 1 | 1 | 1 | 1 | NA | 8 (88.9) |
| 6 | Davari et al. [33] | 1 | 0 | 1 | 1 | 1 | 1 | 1 | 1 | NA | 7 (77.8) |
| 7 | Rasti et al. [34] | 1 | 1 | 0 | 1 | 1 | 1 | 1 | 1 | NA | 7 (77.8) |
| 8 | Shokri et al. [35] | 1 | 0 | 0 | 1 | 1 | 1 | 1 | 1 | NA | 6 (66.7) |
| 9 | Soosaraei et al. [36] | 1 | 1 | 0 | 1 | 1 | 1 | 1 | 1 | NA | 7 (77.8) |
| 10 | Anvari et al. [37] | 1 | 0 | 0 | 1 | 1 | 1 | 1 | 1 | NA | 6 (66.7) |
| 11 | Ahmadi et al. [38] | 0 | 1 | 1 | 1 | 1 | 1 | 1 | 1 | NA | 7 (77.8) |
| 12 | Soleimani et al. [39] | 1 | 0 | 1 | 1 | 1 | 1 | 1 | 1 | NA | 7 (77.8) |
| 13 | Saeidnia et al. [40] | 1 | 1 | 1 | 1 | 1 | 1 | 1 | 1 | NA | 8 (88.9) |
| 14 | Mohammadi et al. [41] | 1 | 1 | 0 | 1 | 1 | 1 | 1 | 1 | NA | 7 (77.8) |
